# Development of a Data-Enabled Mixed Method for Designing for Patients From a Human-Centered Design Perspective: Explorative Case Study

**DOI:** 10.2196/87677

**Published:** 2026-08-19

**Authors:** Yingtao Sun, Jiwon Jung

**Affiliations:** 1 Department of Design, Organisation and Strategy, Faculty of Industrial Design Engineering, Delft University of Technology, Landbergstraat 15, Delft, South Holland, 2628 CE, The Netherlands, 31 152781396; 2 Department of Surgery, Erasmus University Medical Center, Rotterdam, South Holland, The Netherlands; 3 University College, Korea University, Seoul, Republic of Korea

**Keywords:** human-centered design, mixed method, health care design, patient-centered design, data-enabled design, patient journey mapping, patient experience, colorectal cancer, remote patient monitoring

## Abstract

**Background:**

In the early stage of human-centered design (HCD), qualitative and generative methods are commonly used to explore patients’ contexts and needs, emphasizing active patient involvement to ensure that design insights accurately reflect real experiences and enhance both design effectiveness and patient empowerment. However, certain challenges arise in the early stage of the HCD process, including (1) high vulnerability of patient participants, (2) less diverse and representative patient groups due to recruitment challenges, and (3) insufficient problem framing across diverse patient experiences.

**Objective:**

To address these challenges while embracing the system-level HCD perspective, we propose a data-enabled mixed method combining large-scale patient digital research (module A) and in-depth patient engagement research (module B). This paper explores the feasibility and potential value of this mixed method in addressing the identified challenges through a case study.

**Methods:**

In module A, we analyzed a large-scale dataset of online forum posts and validated the extracted topics with medical experts to create a patient community journey map through cocreation sessions. Guided by these findings, module B involved a diary study using a sensitizing paper prototype and semistructured follow-up interviews with 4 patients.

**Results:**

In module A, 37 topics and 10 upper clusters were summarized from 212,107 online posts, revealing that topics associated with the home context exhibited a higher density of emotional content than those related to the hospital context. Patients placed more emphasis on social and mental health during the follow-up stage than in the diagnosis and treatment phases. This shift reveals a gap in current remote monitoring systems, which focus mainly on physical health. Addressing this identified gap, we developed a prototype for use in the module B diary study involving 4 patients. Patients responded positively to the prototype, noting that remote monitoring incorporating social and mental well-being could help them better understand themselves, enhance self-awareness, and improve communication with their physicians. Overall, this study explores the preliminary value of this mixed method in effectively reframing design problems and deeply contextualizing patient needs.

**Conclusions:**

This study provides preliminary evidence supporting the potential of integrating large-scale digital patient research (module A) with in-depth patient engagement (module B) during the early stages of human-centered health care design. The core strength of this mixed method approach lies in its ability to facilitate problem reframing and cultivate a deeper sense of empathy and understanding of patient vulnerability prior to direct engagement. Simultaneously, it captures both the breadth and depth of patient perspectives, offering evidence-based insights that enhance the overall efficacy of user research. Further research across diverse medical contexts is essential to establish the generalizability of these findings.

## Introduction

### Background

Human-centered design (HCD) plays an important role in designing for patients in health care in order to understand patient needs [1], improve patient experience [2], and enhance patient safety [3]. The focus is on empowering designers to understand human needs at a systemic level through empathetic communication and interaction with the individuals within the system [4].

The health care design process typically starts with exploring patients’ needs using the HCD approach before formulating design solutions [5], specifically in the initial stage, such as the discover and define phases of the Double Diamond Model [6] and the fuzzy front end of the design development process [7]. To understand patient context, qualitative research methods are frequently used in the initial stage of the design process to perceive the context [5]. The specific methods proposed in such an explorative stage include both conventional methods, such as interviews and observations, and other innovative methods, such as generative techniques [8], which enable health care designers to deeply explore patients’ needs [9] while also facilitating the examination of the complex interactions among various system components [10].

Central to these methods is involving patients as the end users, serving as a foundational principle for ensuring that insights derived from qualitative research accurately reflect patients’ needs and experiences. For instance, Van Smoorenburg et al [11] asked 10 patients with type 2 diabetes mellitus to fill in sensitizing booklets before conducting interviews to prompt them to reflect on their experiences with self-management of diabetes. Researchers illustrate that the involvement of patients can not only increase the effectiveness and success of the design process [12] but also facilitate patients’ knowledge-building and relationships [13].

### Challenges in Human-Centered Health Care Design

However, implementing HCD and engaging patients in health care settings is more complex than in other domains, primarily due to the intricate nature of health care systems and patients’ compromised health states [14,15]. In this paper, we explore the challenges of designing for patients in the initial stage of the design process, including high vulnerability of patient participants, less diverse and representative patient groups due to recruitment challenges, and insufficient problem framing across diverse patient experiences.

#### High Vulnerability of Patient Participants

Patients are often regarded as a vulnerable demographic, which necessitates careful ethical consideration when involving them in research. For instance, Das and Svanæs [1] noted that discussions about obesity may evoke stigma and shame, requiring designers to plan workshops and participant groupings sensitively to avoid offending others. Furthermore, obtaining medical study approval typically demands a lengthy preparation process, which can be partially attributed to the risk of patients recalling distressing experiences during participation [16]. However, despite the ethical need to protect patients from potential harm, designers are also expected to gain a deep and systematic understanding of their lived experiences to develop meaningful interventions. This requirement poses a notable challenge, as patient participation, despite its recognized value, can introduce burdens that hinder meaningful collaboration [17,18]. Furthermore, designers may not yet be familiar with the patient group in the early design stages, making it challenging to anticipate negative experiences or address such vulnerabilities effectively in advance.

#### Less Diverse and Representative Patient Groups Due to Recruitment Challenges

In the early exploratory phases of HCD, qualitative research methods such as user observations, interviews, and generative techniques are used to emphasize the necessity of continuous patient involvement throughout the entire design lifecycle, rather than limiting participation to a single stage [8,19]. However, these methods face certain dilemmas related to diversity and representativeness of patient groups, which have been criticized as a manifestation of sampling bias in HCD [17]. Specifically, researchers have attributed this challenge to limited sample sizes, difficulties in engaging representative end users, and insufficient participant diversity during patient recruitment, all of which raise concerns about the generalizability, validity, and reliability of the results [15,16,20]. For example, a study developing an intervention with a participatory design approach has reported difficulties in identifying appropriate patient groups and achieving adequate diversity among participants in terms of age, sex, and disease duration [21].

#### Insufficient Problem Framing Across Diverse Patient Experiences

Problem framing is a foundational step in HCD, aiming to ensure that interventions address the right problem [22]. It requires understanding not only users’ needs but also their experiences, values, behaviors, and environmental contexts from a system’s perspective [8]. Nevertheless, the awareness of problem definition in current HCD practices within health care remains limited, with few studies attempting to reframe the initial problem based on diverse user perspectives [23]. More studies adopt a solution-driven rather than a problem-driven approach, generating numerous ideas while leaving the initial design problem insufficiently defined [5]. Moreover, many studies focus on the general principles of HCD rather than detailing specific processes, tools, or methods [24], thereby marginalizing the attention given to defining problems based on patient experiences. For example, some studies fail to clearly distinguish between the discover and define phases in the Double Diamond model [25]. Problem-driven design is essential for prioritizing interventions based on a deep understanding of the underlying problem [5,26]. When patient experiences are explored only within a preestablished problem scope and narrow aspects, rather than through iterative problem reframing, opportunities for creative and efficient solutions are limited [23].

### Advancing the Exploration of Patient Experience With an HCD Perspective: Development of a Data-Enabled Mixed Method

**Figure 1.**
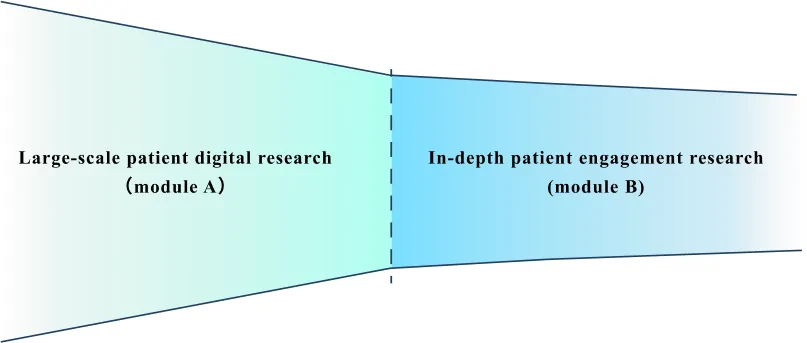
The data-enabled mixed method for designing for patients in the initial stage of the design process.

**Figure 2.**
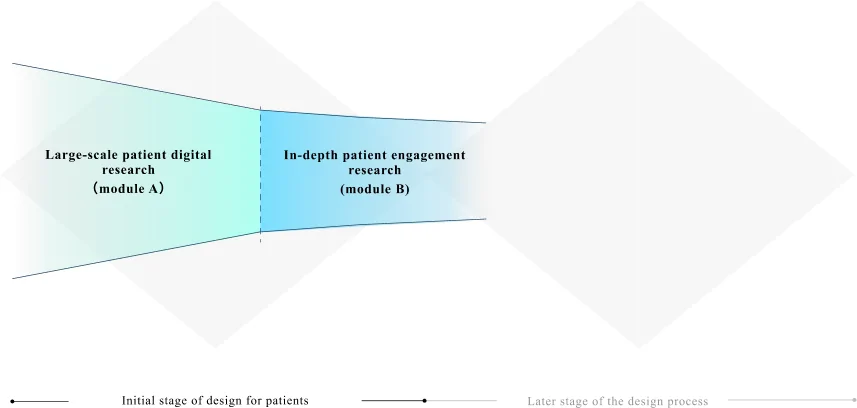
The Double Diamond model as an example illustrates the stage in which the data-enabled mixed method is applied.

To deal with the challenges of designing for patients and achieve the goal of HCD in the early stage, we propose a data-enabled mixed method as illustrated in Figure 1, which prioritizes large-scale patient digital research (module A), subsequently followed by in-depth patient engagement research (module B). It is important to note that we currently consider this mixed method to be most valuable when adopted during the initial stages of the design process, such as the first diamond of the Double Diamond model [6] and the fuzzy front end of the design development process [7]. Figure 2 illustrates the stage in which this mixed method is applied using the Double Diamond model as an example.

In large-scale patient digital research, extensive datasets derived from digital patient data are used to generate meaningful insights. The potential data source can be digital patient platforms such as Facebook groups or patient online forums, which are emerging in patient research [27,28]. In this way, designers can indirectly conduct an in-depth analysis of the data with patients’ perspectives and empathy for patients’ experiences, which further facilitates resonance and the handling of sensitive issues when engaging with patients in the next stage. Patient engagement research in module B refers to conducting conventional qualitative or design research that requires the involvement of patients, such as interviews, diary studies, and focus groups. In other words, we suggest adding large-scale patient digital research to have a generalized understanding before patient engagement in the design process.

### About This Case

An explorative case study illustrates how this data-enabled mixed method was adopted to explore patient experience during the initial stage. The case provides preliminary indications of this method’s potential benefits, including enabling health care designers to acquire rich knowledge that features patients’ perspectives, cultivating an empathetic perspective toward the difficult medical context, facilitating designers to define research and design direction, and designing engaging research activities.

The initial goal of the case was to develop remote patient monitoring for patients with colorectal cancer (CRC) in the Netherlands, considering their quality of life during the follow-up phase. Within this context, this case investigates the care path of patients with CRC with a particular focus on understanding their experiences, especially in the follow-up phase, to lay the groundwork for the development of the remote patient-monitoring system. This study brings together a Netherlands-based interdisciplinary team with expertise in design, computer science, and CRC research.

#### CRC Background and the Theoretical Framework

CRC is the third most common cancer worldwide. It accounts for 10% of the global cancer incidence [29] and is expected to exceed 3 million by 2040 [30]. In the Netherlands, where the case study was conducted, patients who have undergone treatment and surgery for CRC typically enter a 5-year follow-up phase. This phase involves regular meetings between patients and health care professionals to monitor their recovery progress and provide necessary information and support.

**Figure 3.**
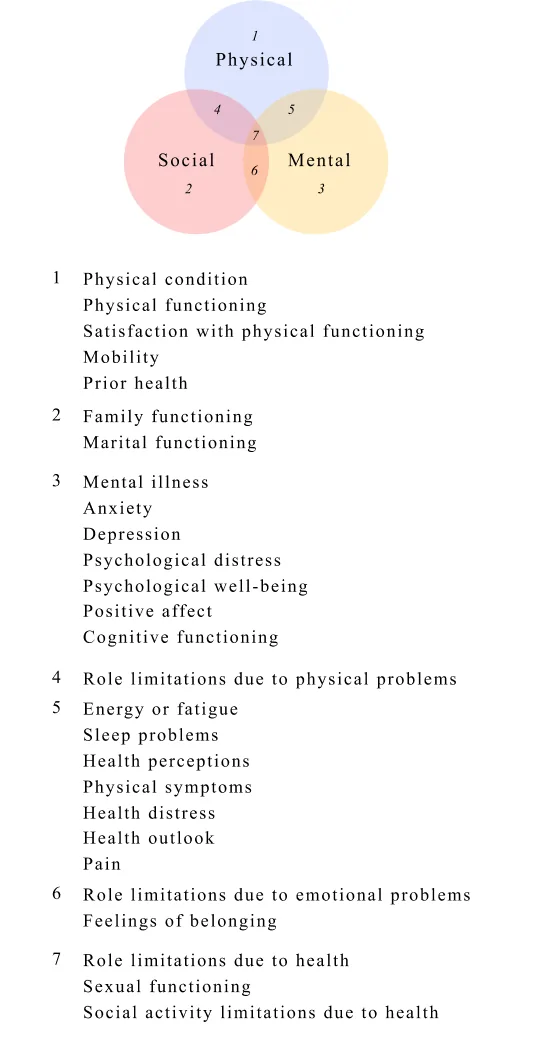
Three-dimensional theoretical framework of health [31].

During the follow-up phase, patients often experience long-term health issues including physical, social, and mental health. The World Health Organization has highlighted the importance of these 3 aspects in relation to health-related quality of life. These 3 aspects are related to each other and form the basis of a framework called “Three-dimensional Theoretical Framework of Health” (Figure 3) [31]. This framework illustrates that the intersection of the 3 aspects has an equally high impact on the quality of life of patients’ health but is often overlooked. It is important to pay attention to all aspects of health of a patient with CRC in the follow-up phase to enhance the patient’s quality of life.

## Methods

### Study Design

**Figure 4.**
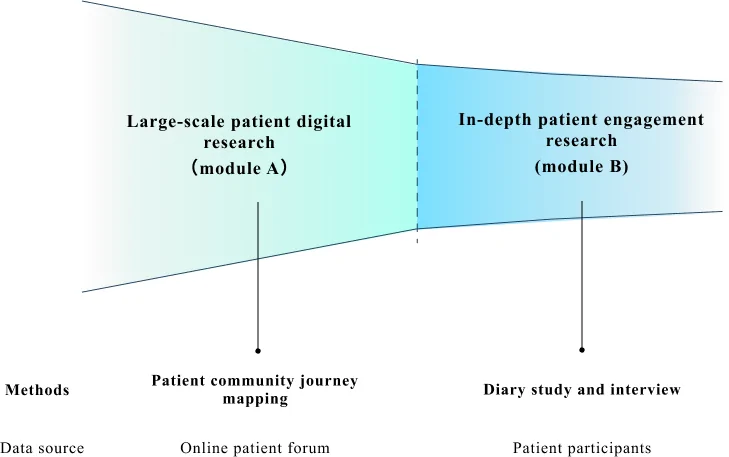
The data-enabled mixed method in the colorectal cancer case.

Figure 4 outlines an overview of the methods used in this case. Module A adopts the patient community journey mapping method developed by Jung et al [32] through the collaboration between the designer and a computer scientist. This method applies natural language processing techniques to online patient posts, analyzing the text and visualizing the results as a journey map [32]. The goal of this method is to perceive the experiences of patients with CRC, reframe the research question, and gain an overview of the patient context. Based on the findings and perceptions from module A, we reframed the research question and then conducted a diary study and interviews involving patient participants to dive deep into the context in module B.

### Module A: Patient Community Journey Map

#### Introduction of Patient Community Journey Map

The conventional development of the patient journey map mainly relies on the data collected from qualitative methods, such as interviews and ethnographic data [33]. Although the qualitative patient journey map reflects a person’s entire journey through the health systems, it requires patient involvement for developing the map and restricts the sample size to at most a few dozen patients [32], bringing the 3 challenges previously mentioned to light. In this case, we adopt the data-enabled “patient community journey mapping” method as one of the examples that can address the 3 challenges and the limitations of the qualitative patient journey map. It takes advantage of natural language processing techniques, such as topic modeling, to process online patients’ stories and further create a journey map with the results [32].

#### Data Collection and Analysis

This study uses the same dataset as our earlier work [34], which introduced the data collection and topic modeling that form an earlier version of module A. Building upon that early-stage exploration, we further investigate the combining of these large-scale digital insights (module A) with in-depth qualitative patient engagement (module B). Additionally, we extend the previous analysis of the patient community journey map by introducing the three-dimensional framework of health to contextualize the findings in module A.

To ensure privacy protection, all data were preprocessed by removing personal identifiers prior to analysis. A total of 212,107 forum posts related to CRC were collected from the publicly accessible Cancer Survivors Network and further preprocessed by applying lemmatization and Term Frequency–Inverse Document Frequency by a computer scientist. The computer scientist then used nonnegative matrix factorization for topic modeling. Because quantitative metrics, such as topic coherence and model stability, consistently declined and lacked clear insights, determining the optimal number of topics relied on rigorous human evaluation. Four researchers, including 2 designers, iteratively reviewed models with 40, 45, and 50 topics, ultimately selecting 50 initial topics as they provided the most diverse and distinctive clusters based on keyword and postexamination. Thirteen topics, such as platform use and expressions of gratitude, were then excluded as they were unrelated to patient experiences, leaving 37 topics for the final analysis.

To analyze the 37 topics, 1 designer reviewed the top 50 posts and top 20 keywords per topic. These were selected by the nonnegative matrix factorization algorithm for possessing the highest dominant topic scores and term weights, respectively, calculated using Term Frequency–Inverse Document Frequency metrics. The review of 50 posts was guided by data saturation: recurring narratives typically emerged after analyzing approximately 30 posts, and the additional 20 were reviewed to rigorously confirm and solidify the extracted themes. The 37 topics were then classified into 10 upper clusters.

After the initial interpretation, a 2-step validation process was implemented to ensure the rigor of the interpretation of the themes. First, the initial interpretation of the 37 topics conducted by the designer was reviewed and validated by a senior design researcher. Second, we subsequently organized cocreation sessions with a panel of local domain experts from the Netherlands. During the 90-minute session, the experts used printed 37 topic cards featuring the top 20 keywords and patient quotes to formulate independent interpretations. These interpretations were then compared with the designer’s initial descriptions for validation. By actively absorbing the experts’ insights and feedback, the team refined the topic descriptions and collaboratively positioned them onto a blank journey map to reflect the 37 topics within the Dutch health care context. This process also ensured that the findings derived from the US-based forum were relevant and contextualized to the Dutch health care system. The expert panel comprised 5 medical professionals: 2 oncological surgeons with 20 and 12 years of experience, respectively; an epidemiologist specializing in patient experience and quality of life with 15 years of expertise; and 2 MD-PhD candidates in surgical oncology with a research focus on CRC. Through these sessions, the expert panel reviewed and validated the identified topics and clusters, ultimately informing the patient community journey map.

After defining the themes, the designer introduced 2 dimensions to classify the topics. The first dimension was to judge whether the topics belonged to a hospital or home context. The second dimension was to decide whether the topics were related to physical, social, or mental health, according to the three-dimensional theoretical framework of health (Figure 3). Additionally, 2 interviews with CRC specialists were carried out to create the journey part of a patient community journey map, visually illustrating patients’ experiences and key concerns.

### Module B: Diary Study and Follow-Up Interview

#### Module Overview

In module A, the research question was reframed: rather than exploring how to develop remote monitoring of patients with CRC during the follow-up stage with a broad consideration of quality of life, the focus was narrowed to specifically supporting patients’ social and mental health. Carrying this new research question forward, module B dives deep into the context of patients with CRC’ by conducting a diary study and interviews on their social and mental health in the follow-up stage when they are at home. The purpose of the study is to understand patients’ social and daily activities and explore patients’ attitudes toward monitoring their activities and emotions.

#### Data Collection and Analysis

In module B, we conducted the diary study and interviewed 4 participants in their follow-up stage. In the diary study, the designer created a paper prototype to simulate remote data collection, aiming to capture patients’ daily activities and emotional states. The content of the prototype was developed based on the 5W1H (What, Why, Who, When, Where, How) structure. By recording the details (what, where, when, who) and their reflections (why, how), patients capture a complete context of their daily lives. The process concludes with patients drawing an emotional curve for the day to visualize their mood trends.

**Figure 5.**
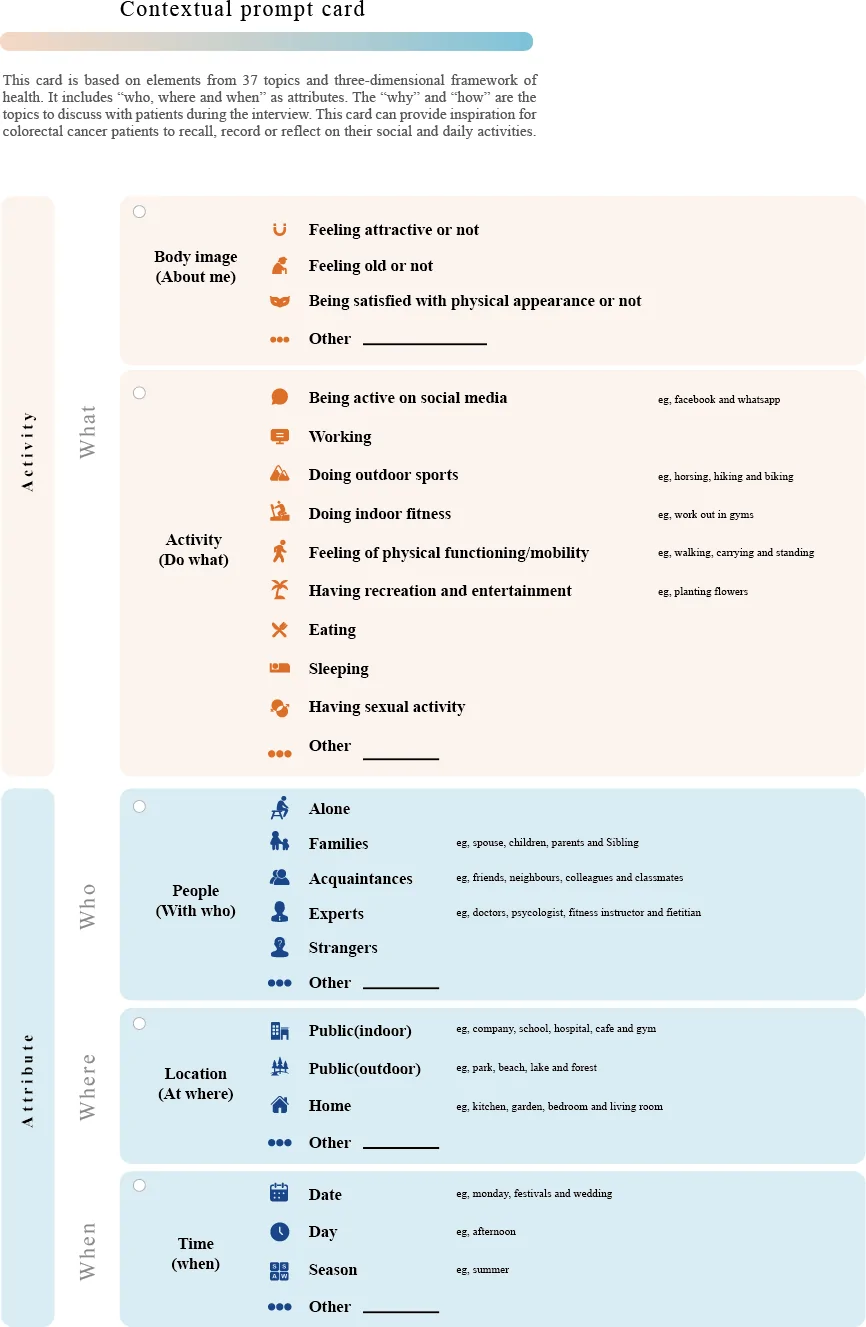
The contextual prompt card was used for patients’ inspiration during the diary study.

In addition, to ensure that the diary study was closely grounded in actual patient experiences, the designer synthesized the 37 topics to create a patient-facing Contextual Prompt Card (Figure 5); for example, the eating activities from topic 17 were in the “What” section in the prompt card. Accompanying the 5W1H paper prototype, this card inspired patients and prevented cognitive overload. It functioned purely as a sensitizing tool, featuring open-ended “Other” options to encourage recording individualized experiences.

Four patients with CRC (Table 1) participated in the study through both online and offline recruitment. Participants were recruited via posters, online forums, and Facebook groups. The patients were invited to finish a 3- to 4-day diary study, followed by a 30-minute semistructured interview to share their findings and attitude.

Although serving as a sensitizing paper prototype rather than independent qualitative data for systematic coding, the diary cards revealed intuitive insights that provided core background for the interviews and acted as observational corroboration in the findings. The formal analytical procedure was exclusively applied to the data collected from the 30-minute semistructured follow-up interviews. We used the thematic analysis consisting of 4 iterative phases. First, descriptive coding was applied to segment the interview text and assign unique codes capturing patients’ direct feedback and daily reflections. Next, through subcategorization, these codes were grouped into specific categories aligned with the interview questions. During the clustering phase, similar codes within each category were aggregated into narrative topics. Finally, theme determination was conducted to categorize related topics and label the final overarching themes.

**Table 1.** Demographic and characteristics of the 4 patient participants with colorectal cancer involved in the diary study and follow-up interviews (module B).

Participant	Age (years)	Year of diagnosis	Number of finished days for diary study	Online or offline interview
Participant A	61	2014	2	Online
Participant B	53	2021	4	Online
Participant C	45	2019	3	Online
Participant D	50	2021	3	Offline (designer visited patient’s home)

### Ethical Considerations

The study was approved by the Human Research Ethics Committee of Delft University of Technology (reference number 2596). Participation was entirely voluntary, with participants retaining the right to withdraw their consent at any time. To guarantee confidentiality and privacy, all collected data were fully anonymized. Participants were assigned unique identification codes, and these deidentified records were stored on secure university servers, accessible exclusively to authorized research members.

## Results

### Module A Results

#### Overview of Module A Results

**Figure 6.**
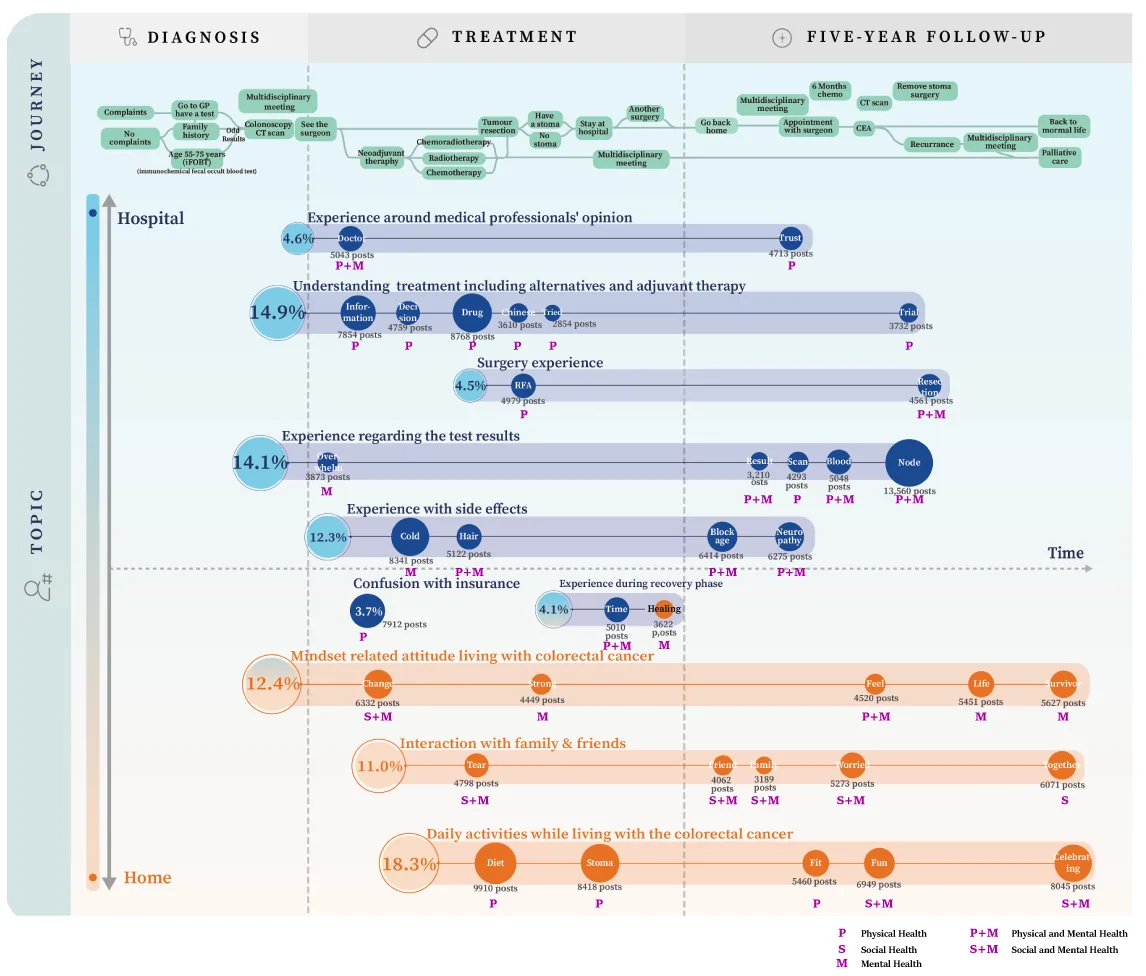
Community journey map of patient with colorectal cancer, with 37 topics classified into 2 dimensions: home and hospital dimension, and three-dimensional framework of health (adapted from Voigt et al [34], which is published under Creative Commons Attribution 4.0 International License [35]) by adding the three-dimensional framework of health [31]. CEA: carcinoembryonic antigen; CT: computed tomography; GP: general practitioner; iFOBT: immunochemical fecal occult blood test; RFA: radio frequency ablation.

Table 2 shows the themes of the 37 topics and 10 upper clusters, and Figure 6 shows the patient community journey map. These topics reflect the broad range of discussions among patients, covering themes from medications and treatments to family life.

**Table 2.** Overview of the 10 upper clusters and 37 topics identified from the large-scale online forum posts (module A) of patient with colorectal cancer (adapted from Voigt et al [34], which is published under Creative Commons Attribution 4.0 International License [35]), with topic numbers added for this analysis.

Cluster number, cluster theme, and topic number	Number of posts under the topic	Topic theme
Cluster 1: Experience around medical professionals’ opinion		
39	5043	Doubts about treatment opinions from medical professionals
45	4713	Suggestion to look for a second medical opinion
Cluster 2: Understanding treatment including alternatives and adjunctive therapy		
30	7854	Patients share their research about alternative therapy options from websites and articles
10	4759	Making treatment decisions for the future with regard to the best outcome and path
33	8768	Listing type, side effect, regimen, and effectiveness of drugs
14	3610	Sharing experience on using Traditional Chinese Medicine to manage health
21	2854	Sharing experiences and recommendations for supplements and medication
48	3732	Sharing information and experiences regarding clinical trials
Cluster 3: Surgery experience		
25	4979	Sharing experience around radio frequency ablation for the liver
23	4561	Sharing negative experience about resection in liver and lung
Cluster 4: Experience regarding the test results (including being worried and confused)		
15	3873	Sharing negative emotions and experiences to live with CRC^a^: overwhelmed, confused, and scared, especially for the tests and screenings
9	3210	Being worried about upcoming scans and results
18	4293	Sharing their outcomes (clear or not) from scanning and caring about how frequently they scan
49	5048	Describing a stressful experience in a blood test; worry about the numbers in the result
8	13,560	Being worried and confused about odd scan results in liver, lung, and lymph
Cluster 5: Experience with side effects		
42	8341	Sharing their experience on managing the side effects of treatment
37	5122	Negative feelings of hair loss due to cancer treatments
34	6414	Feeling uncomfortable due to the obstipation
32	6275	Experiencing pain and numbness due to neuropathy
Cluster 6: Confusion with insurance		
38	7912	Confusion about insurance coverage
Cluster 7: Experience during recovery phase		
27	5010	Describing negative experience of repeated visits to the hospital
20	3622	Staying positive and making life adjustments to their cancer circumstances during the recovery phase
Cluster 8: Mindset-related attitude living with CRC		
0	6332	Difficulties to adjust and adapt to their lives with CRC
41	4449	Sharing how patients can be resilient and positive
24	4520	Sharing their feelings: sick, tired, weak, and bad
46	5451	Sharing their positive philosophical thoughts about living with cancer
11	5627	Survivors sharing their attitudes toward living with cancer along with survival rate
Cluster 9: Interaction with family and friends		
40	4798	Family members’ emotional struggle about having a patient with cancer in their family
47	4062	Experience regarding relationships with friends while having cancer
28	3189	Sharing changes in their family relationship due to cancer journey
43	5273	Being worried about their family members and seeking information on family history (eg, genetic testing)
13	6071	Arguing the importance to spend moments with family members during the cancer journey
Cluster 10: Daily activities while living with the CRC		
17	9910	Sharing suggestions on diets focused on balanced meals and healthy alternatives
22	8418	Suggesting how to take good care of a stoma
35	5460	Sharing experience on being fit again, caring about weight control
31	6949	Sharing ways to stay in a positive mood through planning for distractions
3	8045	Celebrating anniversaries and birthdays for patients as a big milestone of their lives

a CRC: colorectal cancer.

Some topics highlight common concerns among patients with cancer. For example, cluster 1 revolves around sharing apprehensions about doctors’ recommendations and whether seeking a second opinion is necessary. Additionally, under cluster 5, topic 37 indicates the negative feelings of hair loss due to the treatment. Still, some topics go beyond the designer’s initial expectations. To give an example, topic 43 reveals patients’ concerns about the genetic inheritability of cancer and whether their children should undergo genetic testing.

The designer uncovered new insights through reading the posts. Among others, topic 14 indicates that a significant number of patients use Traditional Chinese Medicine to manage their health, and topic 38 reflects patients’ uncertainty about the scope of insurance reimbursement. Additionally, this sensitizing process fostered moments of empathy for the designer. For instance, summarizing topic 40 involved reading numerous posts from family members of patients with cancer, sharing their emotional struggles in coping with a loved one’s illness. Similarly, topic 0 revealed the challenges patients face in adapting to life after being diagnosed.

Through the process of immersing in the posts, the designer gradually perceived the diverse contexts in which these topics occur. Some topics are situated in the hospital context, while others unfold entirely at home. Moreover, these topics span across various health-related categories, including physical, mental, and social health related to the three-dimensional theoretical framework of health [31]. Therefore, we introduced 2 dimensions to the 37 topics, classifying them based on whether they belong to the hospital or home context, as well as by the three-dimensional theoretical framework of health. These topics were visualized into the patient community journey map (Figure 6).

Almost all aspects of health are represented on the map when the topics are examined through the lens of the three-dimensional theoretical framework of health. This indicates that patients with CRC face a wide range of health issues, encompassing most of the categories outlined in Figure 3. The only category not represented is “role limitations due to health,” corresponding to number 7 in the framework shown in Figure 3, which includes aspects such as sexual functioning. We assume that this is because topics related to sexual health are highly private, and consequently, patients may be reluctant to discuss them on public social forums. Nevertheless, this does not imply that these issues are nonexistent but still need attention, as several studies highlight significant challenges in the sexual lives of patients with CRC, such as sexual dysfunction [36-39].

Topics under the hospital context are mostly related to physical health, pathological knowledge, medical procedures, and medical systems. Conversely, topics within the home context primarily revolve around daily life, social interactions, and personal reflections. Patients tend to approach discussions within the hospital context in an objective, rational, and focused manner, while discussions within the home context tend to be more emotionally charged and subjective in their descriptions, aligning with our previous research [34].

In addition, a closer reading of the topics reveals a notable trend: patients’ mental health is frequently influenced by their physical condition. For example, cluster 5 expresses that side effects often cause a lot of physical pain and daily inconveniences, which can be quite stressful for the patient and further affect mental health. This trend is also observed between social and mental health, usually occurring in the home context. For instance, as illustrated in topic 28, patients may experience strong emotional responses to shifts in their social relationships, largely depending on whether these changes are beneficial to them.

Turning to the right side of Figure 6, namely, in the follow-up stage, there is a significant increase in the number of topics in the home context compared with the number of topics associated with the hospital context. Topics such as sharing experience on being fit again (topic 35) appear in this area, demonstrating that patients pay more attention to life in the follow-up stage. As patients become more accustomed to living with their diagnosis, their acute clinical anxiety typically decreases, allowing them to redirect their energy toward social interactions and daily activities. Therefore, social- and mental-related topics are also emerging actively at this stage. Patients gradually become aware of changes occurring in their surroundings. For example, they notice changes in their relationships with friends (topic 47) and illustrate the importance of spending moments with family members (topic 13). These changes are frequently attributed to the impact of cancer and can also lead to emotional fluctuations in the patient. The patients even began to deeply reflect on life, such as being more resilient in the face of fear and death, and share their thoughts with their peers, as observed in topic 46. By contrast, during the diagnosis and treatment phase, patients frequently prioritize attending to their physical well-being, such as being worried about upcoming scans and results (topic 9) and numbers in the result of a blood test (topic 49).

#### Module A Summary

In this case, patient community journey mapping acts as an example to illustrate how large-scale patient research of module A can be conducted. We applied natural language processing techniques to analyze 212,107 posts from patient forums and categorized them into 37 topics. These topics were further classified into 2 dimensions and mapped onto the patient care path.

Since the designer previously lacked knowledge about CRC, this method provides an empathetic and effective starting point, allowing the designer to perceive the context by reading numerous patients’ stories and posts. This indirect dialogue not only contributes empathetic and generalized context to the designer but also allows the designer to have adequate preparation for the patient engagement research in module B.

In module A, the designer not only gained insights into patient experiences throughout the entire care journey but also reframed the research question as follows: How can remote patient monitoring be developed for the follow-up stage with a focus on patients’ social and mental health? This insight emerges from the patient community journey map, echoing that current remote patient monitoring primarily focuses on physical health aspects, such as tracking vital signs while paying relatively little attention to social and mental health. A gap therefore exists, potentially linked to the limited awareness of patients’ social and mental health outside the clinical setting. As a result, it is important to build a remote patient-monitoring system related to social and mental health.

### Module B Results

#### Overview of Module B Results

**Figure 7.**
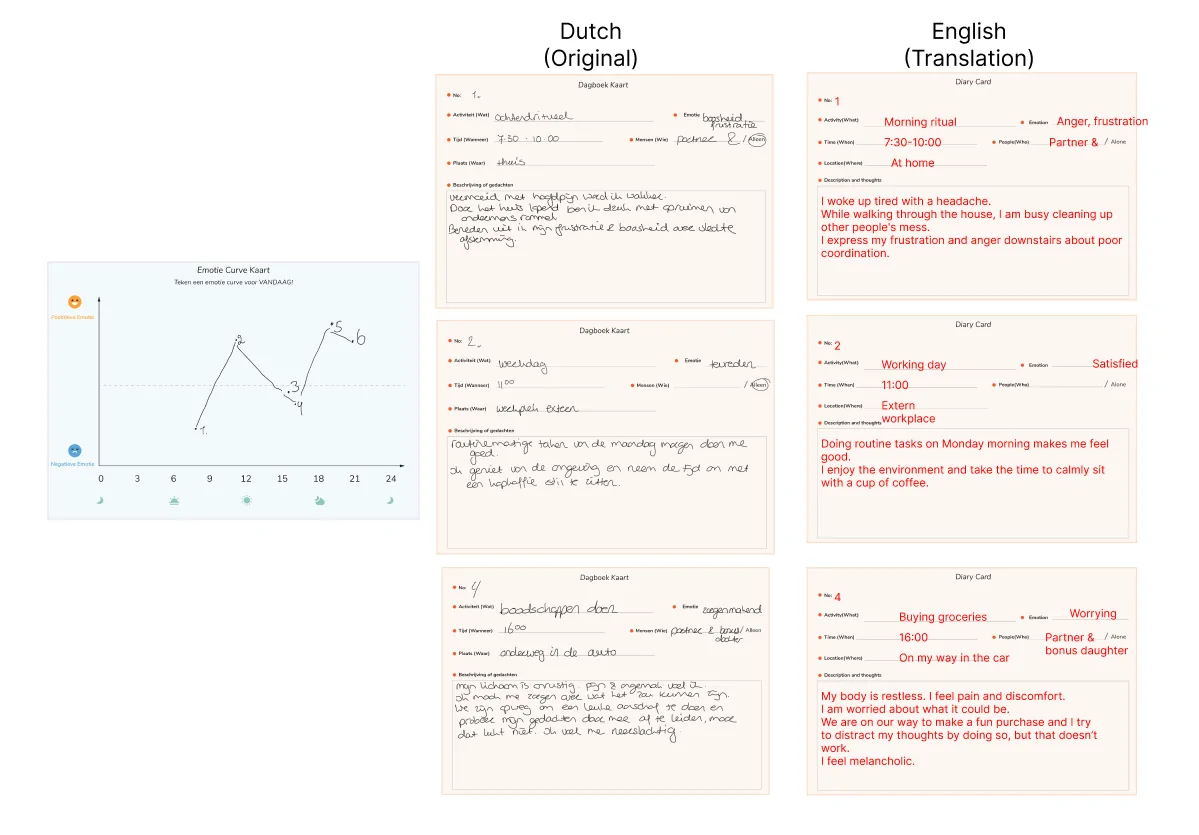
An example of a completed diary card from a patient with colorectal cancer.

The designer observed significant fluctuations in most patients’ emotions throughout the day (Figure 7). These emotional variations are closely linked to specific events or activities. Specifically, emotions are highly correlated with the 5W1H. When patients describe their emotional fluctuations, they often mention the involvement of individuals or the locations where these emotions were experienced. Being alone can also trigger emotional changes in patients, usually associated with past events or experiences, where memories induce emotional responses.

During the study, the designer observed that patients frequently discussed challenges related to their energy levels. Many participants reported experiencing a significant decline in energy following their cancer diagnosis, which restricted their ability to engage in daily activities. For example, participant C expressed that the persistent lack of energy caused considerable frustration:

*I often feel like I want to do something...but it’s very difficult. My body, brain, and mind are not as active anymore...If something takes too much energy, it makes me feel very frustrated*.

[Participant C]

Social engagements are also significantly connected to energy levels. Participants reported that their social circles include more patients, providing mutual support. Patients consider this peer support crucial. However, it also brings certain challenges. Due to their reduced energy levels compared with before their cancer diagnosis, maintaining relationships with old friends, especially those living far away, has become increasingly difficult. As participant D mentioned:

*I’m glad to have friends who are in similar situations as me...and we support each other. However, I also find it difficult to maintain some relationships...I have a good friend who lives a two-hour drive away. In the past, meeting her was easy because driving was not a problem for me, but after the surgery, it has become a bit difficult*.

[Participant D]

Regarding their attitudes toward the prototype and tracking progress, the 4 participants have a very positive attitude. Although 1 patient (participant A) did not complete the study as required, she said that she enjoyed the process very much. The difficulty she found was simply due to the timing, as many years had passed since her CRC diagnosis. “If I had been invited to do this study five years ago, I am sure there would have been very much to record,” explaining her incomplete entries. This also highlights the necessity of early monitoring, given that patients experience much more severe social and emotional fluctuations initially than they do years after diagnosis. Most participants expressed that the process of recording their activities and emotions had heightened their self-awareness and facilitated a deeper understanding of themselves, as the following quotation illustrates:

*It (the prototype) can make you think more about the activities and how you feel about it. For me I am shocked to see how I feel*.

[Participant B]

This self-awareness enables patients to be aware of what activities can have an impact on their mental health:

*It was for me a bad week. I wasn't feeling well and then because of it (prototype) I was very aware that my best friends were my biggest triggers. So I understand more on what happened between them and me*.

[Participant C]

Some patients have reported that the prototype can influence their way of seeing a day and even subsequent behaviors in a positive way:

*When I think I had a bad day, I look back at the diary and I think, oh, it wasn’t so bad. There were just two bad moments...And what this awareness brings as extra is some influence on me...On the third day of the diary study, I realized I didn't have my mindful moment like before which made me realize that it is so important to me, so I'll pick up again*.

[Participant C]

Patients also expressed that they like the prototype more than the questionnaire because it is more personalized and attractive:

*I prefer this one (prototype) because it’s about me*.

[Participant A]

*I really like this...The process is like a story that I can look back on from time to time. I am also tracking myself on diet and weight...*

[Participant D]

When it comes to sharing their data with stakeholders, patients expressed both cautious and positive attitudes. They felt that sharing information about their daily activities and emotions with doctors could help foster a better understanding of their overall well-being. Patients also perceived that health care providers often paid limited attention to their everyday life conditions and emotional states. As a result, these aspects were frequently managed independently by patients, although they had a substantial impact on their health:

*I am willing to share with my doctor, especially when I think I need help. Because I also understand that doctors are very busy...it’s really nice to share your story with other patients that...I woke up and then I got a leakage again. And there is a lot of information I would share with them*.

[Participant D]

#### Module B Summary

Module B used the diary study and follow-up interviews to study 4 patients, mainly to explore the possibility of remote monitoring of activities and emotional well-being in the follow-up phase. Overall, the patients’ attitudes toward the prototype were positive, although there are still several limitations that require further design iterations and refinement. However, the diary study helped us to conduct a preliminary trial of remote patient monitoring and gain a deeper understanding of the patient’s daily life after surgery. The interviews helped us understand the patients’ attitudes and expectations about remote patient monitoring. It also pointed out the possible impacts of remote patient monitoring on patients and key considerations for future implementations. The results of module B are very inspiring for the design of the monitoring system.

## Discussion

### Mixed Methods and the Evolving HCD Context

Emerging research shows the benefits of using mixed methods in patient research, including support for investigating complex health care processes and systems and identifying improvement opportunities [40-42], and the patient data from both qualitative and quantitative research are significant for patient-centered improvement [43]. Despite these benefits, the use of mixed methods in the early HCD process remains underdeveloped, lacking clearly defined best practices and implementation approaches.

Meanwhile, a more system-level perspective in the HCD is required in contemporary practice. Recently, HCD has evolved into 5 principles of humanity-centered design that integrate ecosystems and community perspectives more comprehensively than the 4 principles of HCD, which are solving the core issues, focusing on people, taking a systems point of view, and rapid iterations on interventions [44]. Continually testing and refining proposals is therefore necessary to ensure that they truly meet the needs of the people for whom they are intended. A closer look at this brand-new principle reveals: “Design with the community and as much as possible support designs by the community. Professional designers should serve as enablers, facilitators, and resources, aiding community members to meet their concerns [44].” It requires the designers to take actions not only individually but also from a community or even system level, which corresponds with other research from the view of human-computer interaction [45]. Thus, designers need to understand not only the patients themselves but also the environment and systems around them. It implies that research methods should evolve by combining emerging technologies.

Our experience shows that the mixed method of large-scale research and in-depth research can play an essential role in the beginning of the HCD process. The possible implementation of this method is explored by a case study about the development of the remote patient-monitoring system for patients with CRC. It is important to emphasize that this data-enabled mixed method is not intended to replace traditional qualitative approaches (such as standard interview-only or ethnographic methods), nor do we claim it is universally superior. Rather, it serves as a complementary approach that leverages emerging digital technologies to uncover new possibilities within user research. Compared with other mixed methods, the difference is involving large-scale digital research such as big data analysis in module A, followed by patient engagement research.

### Connection Between Module A and Module B

The connection between module A and module B relies on combining broad and deep analysis, reframing the problem, and translating data into designer empathy.

First, the 2 modules, where module A’s big data results and module B’s in-depth insight, worked as a unified process to ensure both breadth and depth. The large-scale analysis in module A established a macro-level understanding of the persistent social and mental health struggles facing patients with CRC, which acted as a research scope for the entire process. This scope was then brought into sharp focus by module B, where the in-depth engagement with the 4 patients provided the concrete lived experiences that humanized the patterns identified in the big data. During the interviews, the participants’ words did not simply validate the previous findings but extended them by illustrating how these social and mental challenges manifest in daily life. This connection shows that module A provided the necessary generalizability to scope the research, while module B provided the contextual precision to interpret the findings.

Second, module A facilitated the process of problem reframing. Through the analysis of 212,107 posts, our patient community journey map revealed that patients with CRC in the follow-up stage shifted their focus from clinical recovery to social and mental well-being. By contrasting these large-scale digital insights with existing literature, we identified a critical gap: current remote patient monitoring systems overlook the psychosocial dimensions of home-based recovery. This finding directly dictated the scope of the prototype design, shifting it from simple physiological tracking to a system designed to capture daily activities and emotional nuances.

Finally, module A provided a foundational understanding that informed the design of the engagement prototype in module B. An immersion into the patient experience allowed the designer to develop a rich understanding of the participants’ lived realities. By navigating 37 topics and 10 clusters, the designer internalized the emotional landscape of patients with CRC. For instance, when analyzing topic 28, the designer was exposed to the intimate struggles surrounding family relationships and the resulting emotional volatility. This process of deep engagement with patient narratives enabled the designer to move beyond clinical observations and truly resonate with the patients’ underlying anxieties and needs. Consequently, this developed empathy acted as the primary design driver: the designer was moved to incorporate an emotion curve tracking task and specific emotional reflection prompts into the diary study. By transforming these internalized emotional insights into practical research materials, the designer ensured that the engagement activities in module B were designed not just to collect data but to resonate with the patients’ most pressing social and mental health challenges.

### Potential Values of the Data-Enabled Mixed Method

The preliminary findings suggest this data-enabled mixed method has the potential to help health care designers acquire representative insights that reflect patients’ perspectives, develop an empathetic understanding to navigate the complexities of medical contexts, guide the definition of research and design directions, and support the creation of engaging research activities. Together, these capabilities provide critical, informed insights that enhance the effectiveness of patient engagement research. In the following sections, we will illustrate these values in detail by addressing the 3 challenges outlined in the introduction.

#### Addressing the Current HCD Health Care Design Challenges

This case study also provides preliminary evidence that using the data-enabled mixed method has the potential to address the challenges of the current human-centered health care design process involving patients, which are (1) high vulnerability of patient participants, (2) less diverse and representative patient groups due to recruitment challenges, and (3) insufficient problem framing across diverse patient experiences.

The first challenge describes the vulnerability of patient participants. Our data-enabled mixed method tackles this challenge by cultivating designers’ contextual sensitivity prior to patient engagement in the study. In our CRC case, the designer identified sensitive topics, necessary caution in words and behavior, and moments that require alertness before involving them. Meanwhile, the diary study and interviews in module B were more considerate of patients’ vulnerability. For instance, based on insights from module A that patients with CRC often experience fatigue as topic 24 describes, we provided both online and offline interview study options. As shown in Table 1, 3 out of 4 participants chose the online option. For the participant who opted for an offline interview, the designer visited her home for a face-to-face discussion with her consent. Furthermore, as expected, patients also mentioned the impact of declining energy levels on changes in their daily activities and social circles during the interviews. The designer also gained insights into whether patients express positive or negative emotions when discussing these topics. Particularly when patients presented certain topics with highly negative emotions, the topics were given special attention during the interview process. This enables the designer to avoid language and actions that may cause discomfort or be sensitive to patients, or at the very least, have expectations and preparations for the upcoming situation. The respect for patients aligns with the proposal by Das and Svanæs, referring to “Particular considerations and facilitation is required when including vulnerable user groups in human-centered design activities [1].”

The second challenge, related to less diverse and representative patient groups due to recruitment challenges, can be effectively addressed by the combination of modules A and B. The large dataset of 212,107 posts given in module A contributed to mitigating the lack of representativeness caused by small samples [46]. The 37 topics related to patient experiences during their care path have been identified from these posts, with thousands of posts associated with each topic. Furthermore, we realized that many of these topics align with the ones discussed during the patient engagement studies in module B. For example, participant D illustrates the mutual support shared among patient peers, which also reflects in patient posts of sharing strategies for coping and improving quality of life during the follow-up stage. However, some of the topics found in module A were more detailed and did not show up in module B. For example, topic 43 revealed patients’ concerns about the potential hereditary nature of CRC and topic 3 highlighted the significance of anniversaries and birthdays to patients. These findings, which were not evident in the diary study, yet represent intriguing new insights. These representative outcomes provide shared insights from different demographic patient groups and can be applicable to a wider system.

The third challenge concerns the reframing of problems from the patient perspective during the early stages of HCD. The potential of our method in addressing this challenge has been discussed in the previous section (Connection Between Module A and Module B), where we highlighted that conducting large-scale patient research can help reframe research and design problems. If module A had not been conducted and a more solution-driven approach had been adopted instead, the focus might have been on features such as information registration, symptom monitoring, patient education, and personalized advice. Consequently, the social and mental health needs of patients might remain overlooked or fail to emerge as explicit areas of concern. Our method encourages designers to define research and design questions based on the diverse and in-depth perspectives of patient voices. For instance, designers can easily learn that patients with CRC need to undergo blood tests to monitor treatment effectiveness. However, it was only through topic 49 in module A that the designer realized that the numerical results of these tests can cause emotional instability for a large number of patients. The rich context obtained from the large-scale data can not only identify problems within complex health systems [47] but also contribute to designing desirable experiences [48].

### Limitations

Naturally, this data-enabled mixed method has certain limitations. From the perspective of organizations, designers may lack proficiency in handling large-scale data. The data processing process would require the involvement of computer scientists. Although interdisciplinary collaboration is beneficial to the design process, in practice user research in HCD itself has been proven to be time-consuming and costly [49], and collaboration with computer scientists may exacerbate this phenomenon. It is therefore necessary to explore further how to effectively work together with data scientists. While the vastness of the data helps obscure individual identities, ethical and privacy concerns still demand rigorous attention. When collecting raw data, researchers must selectively gather information to ensure complete anonymization.

In addition, a notable limitation of this case study is the selection bias inherent in using English-language online forums, which excludes those with limited digital literacy or non-English proficiency. To mitigate this, we engaged local domain experts from Rotterdam, an area with a high concentration of underserved and diverse patient populations, and further enriched our data through interviews with Dutch-speaking patients in module B. However, due to the small sample size in these engagement phases, our findings may still underrepresent marginalized or offline populations. Future research should incorporate multilingual, multiplatform datasets and larger, more diverse cohorts to enhance generalizability.

Another potential limitation of this case study is that relying on a single designer restricts the capacity for independent cross-validation. While the expert cocreation sessions in module A helped mitigate this risk, future applications should ideally involve a larger interdisciplinary team to independently cross-analyze the modules, thereby further minimizing subjective bias and enhancing overall reliability.

Although the proposed approach was empirically applied to the specific context of CRC, the results indicate preliminary feasibility rather than definitive proof of the method’s applicability across diverse health care domains, care paths, or varying team structures. Additionally, the small sample size in module B (n=4) restricts the generalizability of the qualitative findings in this case, precluding a comprehensive evaluation of the prototype’s efficacy or definitive design requirements. However, the primary objective of this study was to explore the feasibility and values of the methodological pipeline. Within this scope, the small-scale engagement shed light on preliminary insights into how qualitative interactions can contextualize, ground, and humanize the broad insights derived from the massive data in module A, ultimately addressing the challenges encountered by health care designers in the initial design stage.

### Future Research

Building upon the limitations identified in this exploratory case study, future work should prioritize rigorously validating the method’s generalizability and establishing best practices. Since our current findings represent preliminary indications based on a CRC case, future comparative studies and multiple case studies across diverse health care domains and different disease care paths are required to establish robust evidence.

Furthermore, to mitigate the selection bias inherent in online datasets, subsequent applications of this data-enabled mixed method should incorporate multilingual datasets from diverse geographical platforms. During the engagement research phase (module B), researchers should also explore the specific contexts of the participants, particularly investigating whether their situations reflect the experiences of marginalized populations, such as non-English speakers or individuals with limited digital access.

In addition to diversifying the data platforms, as longitudinal datasets are increasingly integrated into this process, future studies using this data-enabled mixed method should consider and validate the temporal insights to ensure that they reflect current state-of-the-art practices. To explain this within the context of our case, researchers using longitudinal online datasets must be mindful of the temporal dimension. This caution is necessary because while certain patient experiences, such as psychosocial challenges, often remain consistent over time, specific medical contexts, such as treatment modalities, surgical advancements, or evolving care protocols, can undergo rapid technological shifts.

While this method has initially centered on patients, future research should expand to include other stakeholders, such as caregivers and health care professionals, thereby fostering a more holistic design approach. In parallel with expanding the participant scope, future practices could also explore methodological flexibility. For instance, although we currently recommend conducting module A before module B, researchers might alternate this sequence by using large-scale digital research to validate prior in-depth qualitative findings. Ultimately, to support these methodological advancements, integrate emerging technologies, and mitigate the risk of confirmation bias, continuous interdisciplinary collaboration among health care designers, data scientists, and health care professionals is essential. Such team-based approaches will enable cross-validation of the results in both modules and establish standardized, objective best practices.

### Conclusions

This paper highlights the trend of HCD adopting a more system-level perspective, which introduces challenges for health care designers at the early stages of patient-focused design, including (1) high vulnerability of patient participants, (2) less diverse and representative patient groups due to recruitment challenges, and (3) insufficient problem framing across diverse patient experiences. To address these challenges, we propose a data-enabled mixed method combining large-scale digital patient research (module A) and in-depth engagement research (module B).

We explore this approach through a case study of CRC follow-up care, using patient community journey mapping (module A) and diary studies and interviews (module B) to inform the development of a remote patient-monitoring system.

Based on this specific case study, there are preliminary indications that this method has the potential to support problem reframing and cultivate empathy toward patient vulnerability before conducting engagement research. By capturing the diversity and depth of patient voices within this context, it generated evidence-based insights that appeared to enhance the design of subsequent patient engagement activities. While these initial findings are promising, future research across broader health care contexts is needed to generate generalizable evidence.

## Abbreviations

CRC: colorectal cancer
HCD: human-centered design
